# Blockage of transient receptor potential vanilloid 4 inhibits brain edema in middle cerebral artery occlusion mice

**DOI:** 10.3389/fncel.2015.00141

**Published:** 2015-04-10

**Authors:** Pinghui Jie, Yujing Tian, Zhiwen Hong, Lin Li, Libin Zhou, Lei Chen, Ling Chen

**Affiliations:** Department of Physiology, Nanjing Medical UniversityNanjing, China

**Keywords:** cerebral ischemia, transient receptor potential vanilloid 4, brain edema, matrix metalloproteinase, tight junction protein

## Abstract

Brain edema is an important pathological process during stroke. Activation of transient receptor potential vanilloid 4 (TRPV4) causes an up-regulation of matrix metalloproteinases (MMPs) in lung tissue. MMP can digest the endothelial basal lamina to destroy blood brain barrier, leading to vasogenic brain edema. Herein, we tested whether TRPV4-blockage could inhibit brain edema through inhibiting MMPs in middle cerebral artery occlusion (MCAO) mice. We found that the brain water content and Evans blue extravasation at 48 h post-MCAO were reduced by a TRPV4 antagonist HC-067047. The increased MMP-2/9 protein expression in hippocampi of MCAO mice was attenuated by HC-067046, but only the increased MMP-9 activity was blocked by HC-067047. The loss of zonula occludens-1 (ZO-1) and occludin protein in MCAO mice was also attenuated by HC-067047. Moreover, MMP-2/9 protein expression increased in mice treated with a TRPV4 agonist GSK1016790A, but only MMP-9 activity was increased by GSK1016790A. Finally, ZO-1 and occludin protein expression was decreased by GSK1016790A, which was reversed by an MMP-9 inhibitor. We conclude that blockage of TRPV4 may inhibit brain edema in cerebral ischemia through inhibiting MMP-9 activation and the loss of tight junction protein.

## Introduction

Brain edema, defined as an increase of brain tissue volume resulting from an increase in its fluid content, is an important pathological process in many central nervous system diseases, such as cerebral ischemia, traumatic brain injury, and epilepsy. The brain edema observed in the cerebral ischemia has a characteristic time course and it begins with a cytotoxic phase, due to the intracellular fluid accumulation caused by energy failure after cessation of the cerebral circulation (Gotoh et al., 1985; Song and Yu, 2014). With ongoing reperfusion, the secondary vasogenic edema develops due to the disruption of blood brain barrier (BBB) (Gotoh et al., 1985; Song and Yu, 2014). During ischemia-reperfusion, brain edema is an important pathological process and related to unfavorable outcomes (Gotoh et al., 1985; Song and Yu, 2014). Expression of matrix metalloproteinase (MMP) in healthy brain is relatively low, but activated MMPs, especially MMP-2 and MMP-9, increase during stroke, which leads to disruption of blood brain barrier (BBB) integrity and contributes to brain edema (Machado et al., 2006; Rosenberg and Yang, 2007; Lenglet et al., 2014). Blocking MMP activation has favorable effect in preventing cerebral damage after cerebral ischemia (Cui et al., 2012; Guo et al., 2014; Lee et al., 2014).

Transient receptor potential vanilloid 4 (TRPV4), a member of transient receptor potential superfamily, is permeable to calcium (Ca^2+^) (Garcia-Elias et al., 2014). TRPV4 can be activated by diverse stimulation, including cell swelling-induced mechanical stimulation, modest heat, hypotonic stimulation, endogenous and synthetic ligands (Vincent and Duncton, 2011). Our recent study reveals that the protein level of TRPV4 increases during ischemia-reperfusion (Jie et al., 2014). Combined with the characteristic of multipolar sensitivity, TRPV4 has been proven to be involved in cerebral ischemic neuronal injury (Lipski et al., 2006; Bai and Lipski, 2010; Li et al., 2013; Jie et al., 2014). BBB permeability is increased by activation of TRPV4 and TRPV2 complex (Brown et al., 2008). In lung tissues, application of TRPV4 agonist increases the level of activated MMPs (Villalta et al., 2014). Whether TRPV4 activation could increase MMP activity in brain tissue remains unclear. In this study, we first examined whether blockage of TRPV4 could reduce brain edema in a mouse model of focal cerebral ischemia by using the middle cerebral artery occlusion (MCAO) and then explored the mechanism underlying TRPV4 action by examining the effect of TRPV4 blockage/activation on the expression and activity of MMP-2/9.

## Materials and methods

### Subjects

Male mice (ICR, Oirental Bio Service Inc., Nanjing, China), weighing 25–30 g, were used. All experimental procedures were conformed to Guidelines for Laboratory Animal Research of Nanjing Medical University and were approved by Institutional Animal Care and Use Committee of Nanjing Medical University. Each experimental group contained nine mice.

### Drug treatment

Drugs were intracerebroventricularly (icv.) injected as previously reported (Jie et al., 2014). In brief, the mice were placed in a stereotaxic device (Kopf Instruments, Tujunga, CA, USA) after they were anesthetized. For a single icv. injection of GSK1016790A, a 26-G stainless steel needle (Plastics One, Roanoke, VA) was inserted into the lateral ventricle (0.3 mm posterior, 1.0 mm lateral, and 2.5 mm ventral to the bregma). For repeated icv. injections of HC-067047, a 23-G stainless steel guide cannula (Plastics One, Roanoke, VA) was first implanted into the right lateral ventricle and anchored to the skull with stainless steel screws and dental cement. HC-067047 was injected beginning at 4 h after MCAO and then injected every 8 h until 48 h post-MCAO using a 26-G stainless steel needle. GSK1016790A at the dose of 1 μ M/mouse was chosen because it causes significant hippocampal neuronal damage as well as the changes in protein levels of phosphrylation of Akt and ERK (Jie et al., 2014). HC-067047 at the dose of 10 μ M/mouse was used because it reduces the brain infarction at 24 h post-MCAO (Li et al., 2013). SB-3CT is the selective gelatinase inhibitor and can inhibit MMP-9 activity *in vivo* (Gooyit et al., 2012). Here, SB-3CT (25 mg/kg) was intraperitoneally injected 30 min before GSK1016790A-injection and then injected once daily for 3 days. This dose was used because it antagonized the increase of activated MMP-9 and exerted neuroprotection in transient focal cerebral ischemia (Cui et al., 2012). Control mice were injected with same volume of vehicle.

### Preparation of focal cerebral ischemia model

Transient focal cerebral ischemia was induced by MCAO as previously described (Li et al., 2013). Sham-operated animals were treated identically, except that the middle cerebral artery was not occluded after neck incision.

### Analysis of tissue water content

The extent of post-ischemic brain edema was determined by the wet- and dry-weight (wt) at 48 h post-MCAO. After the mice were decapitated under anesthesia, the olfactory bulb, cerebellum and brainstem were removed. Left (contralateral) and right (ipsilateral) hemispheres were separated and then the wet weight was determined immediately. The dry weight was determined after drying the tissue to a constant weight at 100°C. Tissue water content was calculated as % H_2_O = (1-dry wt/wet wt)^*^100%.

### Assessment of Evans blue (EB) extravasation

2% EB (4 ml/kg) was injected via the tail vein at 46 h post-MCAO and the mice were perfused under anesthesia at 48 h post-MCAO. The cerebral hemispheres were collected, followed by homogenization with 50% trichloracetic acid and centrifugation. The amount of EB in the cerebral hemispheres was quantified at 620 nm by spectrofluorophotometry as previously described (Wang et al., 2014). EB leakage of each sample was quantified using a standard curve.

### Western blot analysis

Western blot analysis was performed at 48 h post-MCAO or on day 3 after GSK1016790A-injection as reported (Li et al., 2013; Jie et al., 2014). Primary antibodies against MMP-2 (1:1000), MMP-9 (1:1000) and glyceraldehyde-3-phosphate dehydrogenase (GAPDH) (1:5000) were obtained from Abcam and those against ZO-1 (1:1000) and occludin (1:1000) were from Cell Signaling Technology. Hippocampal samples were collected from the hemisphere of three mice as a set of western blot analysis. The summarized data represent the average of three experimental sets.

### Gelatin zymography

At 48 h post-MCAO or on day 3 after GSK1016790A-injection, the mice were perfused transcardially with ice-cold phosphate-buffered saline under anesthesia. The hippocampi were dissected, frozen immediately on dry ice, and stored at −80°C. Brain samples were homogenized in lysis buffer including protease inhibitors on ice. After centrifugation, the supernatants were collected, and total protein concentration was determined using the Bradford assay (Bio-Rad, Hercules, CA, USA). Prepared protein samples were loaded and separated by 10% Tris-glycine gel with 0.1% gelatin as substrate. After separation by electrophoresis, the gel was renaturated and then incubated with developing buffer at 37°C for 24 h as described previously (Lee et al., 2008). After developing, the gel was stained with 0.5% Coomassie Blue R-250 for 30 min and then destained appropriately. Proteolytic bands in the zymography were quantified by scanning densitometry (Quantity one, Bio-Rad).

### Data analysis

Data are expressed as means ± S.E.M and analyzed with Stata 7.0 software (STATA Corporation, USA). The EB leakage, protein levels and MMP-2/9 activity in MCAO mice or HC-067047-treated sham-op mice or MCAO mice was normalized to those in sham-op mice. Protein levels and MMP-2/9 activity in the mice injected with GSK1016790A or/and SB-3CT was normalized to those in the vehicle-injected mice (control mice). ANOVA followed by Bonferroni's *post-hoc* test was used for statistical analysis, and significance levels were set at *P* < 0.05 and *P* < 0.01.

## Results

### Effect of TRPV4 antagonist on the brain water content and EB extravasation in MCAO mice

**Figure 1:**
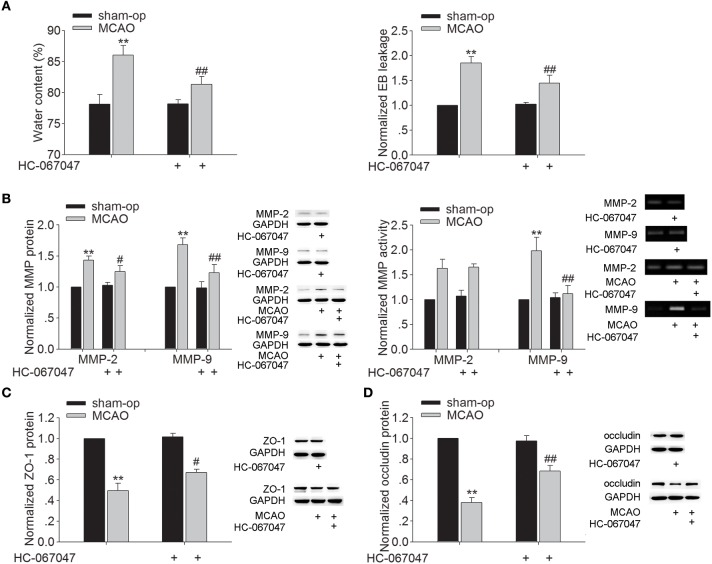
**Effect of TRPV4 antagonist on brain water content, EB extravasation, the protein levels and activity of MMP-2/9, and the protein levels of ZO-1 and occludin at 48 h post-MCAO**. **(A)** Bar graphs show the brain water content and EB extravasation in the ipsilateral hemisphere in sham-op, MCAO, and HC-067047-treated MCAO mice. **(B)** The protein levels of MMP-2 and MMP-9 increased at 48 h post-MCAO, which was sensitive to HC-067047 treatment. By contrast, only the increased activity of MMP-9 was markedly blocked by HC-067047 treatment. **(C,D)** The protein levels of ZO-1 **(C)** and occludin **(D)** decreased at 48 h post-MCAO, which was markedly reversed by HC-067047 treatment. ^**^P < 0.01 vs. sham-op, ^#^*P* < 0.05 and ^##^*P* < 0.01 vs. MCAO mice.

Brain edema began to develop as early as 3 h after MCAO and progressed rapidly to a maximum on the second and third day, and gradually regressed thereafter (Gotoh et al., 1985). Therefore, we examined the effect of HC-067047, a specific TRPV4 antagonist, on the brain water content at 48 h post-MCAO. As shown in Figure 1A, the brain water content of ischemic ipsilateral hemisphere was 86.03 ± 1.55% in MCAO mice. After treatment with HC-067047, the water content of ipsilateral hemisphere was reduced to 81.36 ± 1.23% (*P* < 0.01). EB extravasation in the ipsilateral hemisphere was increased at 48 h post-MCAO. A significant decrease of EB extravasation in the ipsilateral hemisphere was found when the MCAO mice were treated with HC-067047 (*P* < 0.01). Application of HC-067047 had no effect on the water content or EB leakage in the contralateral hemisphere (*P* > 0.05, data not shown). The above results indicate that blockage of TRPV4 inhibits the formation of brain edema following ischemic insult.

### Effect of TRPV4 antagonist on the protein expression and activity of MMP-2/9 and the protein expression of ZO-1 and occludin in MCAO mice

During stroke, MMP-2 and MMP-9 are up-regulated, leading to increased BBB permeability through degradation of endothelial matrix proteins. Figure 1B shows that protein levels of MMP-2 and MMP-9 markedly increased at 48 h post-MCAO, and this increase was attenuated by HC-067047 (*P* < 0.05 for MMP-2, *P* < 0.01 for MMP-9). Moreover, an increase of MMP-2 and MMP-9 activity was found at 48 h post-MCAO, but only the increase of MMP-9 activity was markedly inhibited by HC-067047 treatment (*P* < 0.01). We then studied the effect of TRPV4-blockage on the expression of zonula occludens-1 (ZO-1) and occludin, two major proteins involved in the tight junctions of BBB. Figures 1C,D show that protein levels of ZO-1 and occludin decreased markedly at 48 h post-MCAO, which was rescued by the treatment with HC-067047 (*P* < 0.05 for ZO-1, *P* < 0.01 for occludin). These results indicate that blockage of TRPV4 could inhibit the activated MMP-9 level and rescue the loss of ZO-1 and occludin in MCAO mice, which may benefit for the integrity of BBB.

### Effect of TRPV4 agonist on the protein expression and activity of MMP-2/9 and the protein expression of ZO-1 and occludin

**Figure 2:**
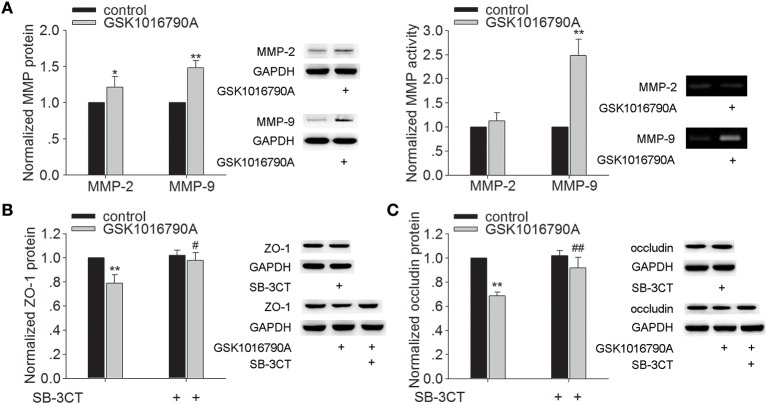
**Effect of TRPV4 agonist on the protein levels and activity of MMP-2/9 and the protein levels of ZO-1 and occluding**. **(A)** The protein levels of MMP-2/9 increased significantly after icv. injection of GSK1016790A, but only the activity of MMP-9 increased markedly after GSK1016790A treatment. **(B,C)** Lower protein levels of ZO-1 **(B)** and occludin **(C)** were found in GSK1016790A-injected mice, which was sensitive to a MMP-2/9 inhibitor. ^*^P < 0.05 and ^**^P < 0.01 vs. control, ^#^*P* < 0.05 and ^##^*P* < 0.01 vs. GSK1016790A-injected mice.

As shown in Figure 2A, the protein levels of MMP-2 and MMP-9 were higher in the mice injected with GSK1016790A (a TRPV4 agonist), when compared to control values (*P* < 0.05 for MMP-2, *P* < 0.01 for MMP-9). It was noted that only the activity of MMP-9 was increased markedly by GSK1016790A treatment (*P* < 0.01). Protein levels of ZO-1 and occludin were significantly lower in GSK1016790A-injected mice, compared to control levels (*P* < 0.01 in each case) (Figures 2B,C). It was noted that higher protein levels of ZO-1 and occludin were found in the mice co-injected with GSK1016790A and SB-3CT (an MMP-9 inhibitor) (*P* < 0.05 for ZO-1, *P* < 0.01 for occludin). These results indicate that activation of TRPV4 may facilitate the activation of MMP-9 to decrease the protein of ZO-1 and occludin.

## Discussion

During the cerebral ischemica, cytotoxic cell swelling develops shortly after the occlusion of cerebral artery and is caused by energy failure. It is the earliest morphological change that cannot be avoided, but this cellular morphology change indeed facilitates the activation of TRPV4. Vasogenic edema is a secondary phase of brain edema, resulting from BBB breakdown (Gotoh et al., 1985; Song and Yu, 2014). Here, application of HC-067047 decreased the water content and EB leakage in the ipsilateral hemisphere of MCAO mice (Figure 1A) and this effect of closing TRPV4 is more likely due to the inhibition of vasogenic edema.

MMP is a family of zinc-dependent endopeptidases. Among them, MMP-2 and MMP-9 are major MMPs that are involved in BBB injury and neuronal death after cerebral ischemia (Machado et al., 2006; Rosenberg and Yang, 2007; Lenglet et al., 2014). *In vitro* and *in vivo* studies have proven that blocking MMP activation is a potential therapeutic strategy for preventing neuronal death through preserving BBB integrity following ischemic brain injury (Machado et al., 2006; Fu et al., 2014). Here, the increase of MMP-9 protein expression and activity at 48 h post-MCAO was markedly blocked by TRPV4 antagonist. Consistently, MMP-9 protein level and activity in hippocampus were higher in GSK1016790A-injected mice. Although the protein level of MMP-2 was increased by application of TRPV4 agonist, the activity was nearly unaffected (Figures 1B, 2A). Here, we also found that GSK1016790A-induced modulation of MMP-9 protein expression and activity was blocked by application of TRPV4 antagonist HC-067047 (Supplementary Figure 1). Therefore, MMP-9 is more likely the target of TRPV4. Recent studies show that the treatment with TRPV4 agonist leads to an increase of activated MMPs (MMP-1, MMP-2 and MMP-9) in lung tissues, which is dependent on TRPV4-mediated Ca^2+^ influx (Li et al., 2011; Villalta et al., 2014). The pathological changes in a stroke may facilitate the activation of TRPV4 and moreover, an increased TRPV4 protein level is found during ischemia-reperfusion (Jie et al., 2014). Besides the Ca^2+^ entry mediated by TRPV4, activation of TRPV4 enhances NMDA-activated current (Li et al., 2013). Therefore, it is proposed that during stroke, the over- or hyper-activation of TRPV4 aggravates the increase of [Ca^2+^]_i_, helping to increase the activated MMP-9.

The BBB is formed by brain endothelia cells lining the microvascular system that is sealed by tight junction (TJ). TJ is crucial for the permeable property of BBB. ZO-1 and occludin are two major proteins involved in TJ of BBB (Salama et al., 2006). ZO-1 is a peripheral membrane protein and considered an indicator for the presence of TJ. Occludin interacts with ZO-1, which in turn, links TJ to the cytoskeleton. Here, a decrease of ZO-1 and occludin protein was found in GSK1016790A-injected mice (Figures 2B,C) and the loss of ZO-1 and occludin protein at 48 h post-MCAO was markedly blocked by HC-067047 (Figures 1C,D). Activation of MMP opens the BBB by degrading TJ and increases BBB permeability after stroke, and an MMP inhibitor prevents the degradation of TJ and attenuates BBB disruption (Cui et al., 2012). The present study found that GSK1016790A-induced decrease of ZO-1 and occludin protein was significantly reserved by an MMP-9 inhibitor (Figures 2B,C). Moreover, GSK1016790A-induced the loss of ZO-1 and occludin was completely blocked by HC-067047 (Supplementary Figure 1). Our data suggested that during cerebral ischemia, activation of TRPV4 may increase the activated MMP-9 level, leading to the loss of ZO-1 and occludin. In the future study, TRPV4 knockout mice will be needed to further clarify TRPV4-induced the above action and its involvement in the brain edema formation during cerebral ischemia.

## Conclusion

Brain edema, either a cause or a consequence, disturbs physiological neuronal function and amplifies tissue damage. Together with our early reports (Li et al., 2013; Jie et al., 2014), the present data indicate that during cerebral ischemia, blockage of TRPV4 may inhibit the activation of MMP-9 and the loss of ZO-1 and occludin, which helps to reduce the disruption of BBB integrity and the subsequent vasogenic brain edema and brain injury. Our unpublished data find that application of TRPV4 agonist decreases the protein of claudin-5 in hippocampus, indicating that, besides ZO-1 and occludin, activation of TRPV4 may result in the loss of other TJ protein. As is known that aquaporin-4 (AQ4) plays an important role in the formation of brain edema during stroke. TRPV4 and AQ4 co-expressed in the astrocytic endfeet and it acts as an important structural and functional partner of AQ4 (Benfenati et al., 2011). Therefore, TRPV4 is likely a potential target for treating brain edema during cerebral ischemia and other pathological process.

### Conflict of interest statement

The authors declare that the research was conducted in the absence of any commercial or financial relationships that could be construed as a potential conflict of interest.

## Supplementary material

Supplementary Figure 1 **Effect of HC-067047 on GSK1016790A-induced modulation on MMP-9 and TJ**. TRPV4 antagonist HC-067047 (10 μ M/mouse) was icv. injected 30 min before GSK1016790A injection, and then HC-067047 was injected once daily for the 3 days. GSK1016790A-induced the increase of MMP-9 protein level and activity **(A)**, and the decrease of ZO-1 and occludin protein levels **(B)** were blocked completely by HC-067047. ^**^*P* < 0.01 vs. control and ^##^*P* < 0.01 vs. GSK1016790A-injected mice.
