# Supplementary figures and images for: Blockage of transient receptor potential vanilloid 4 inhibits brain edema in middle cerebral artery occlusion mice
Source: Front Cell Neurosci. 2015 Apr 10;9:141. doi: 10.3389/fncel.2015.00141 (PMC4392311; doi:10.3389/fncel.2015.00141)

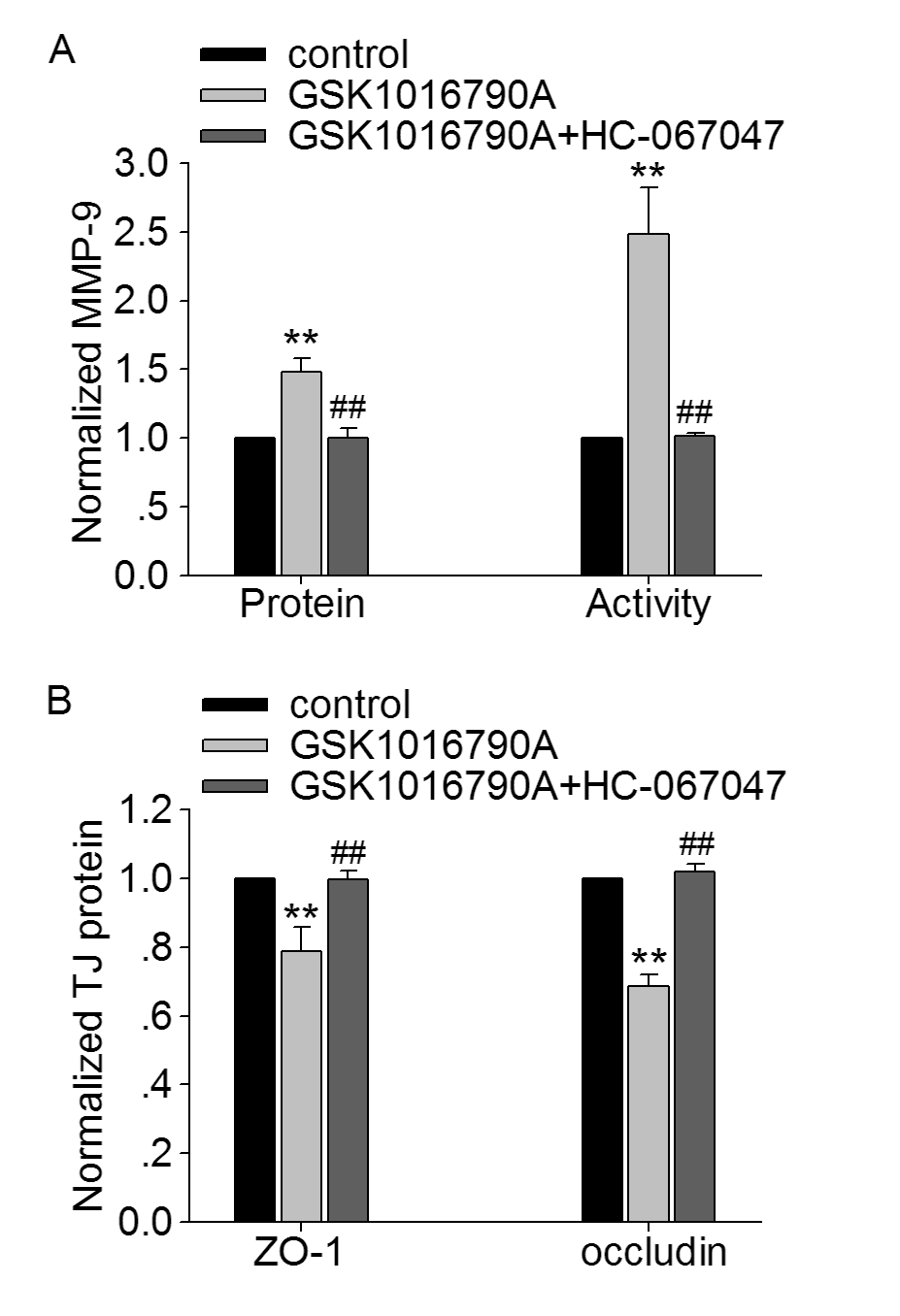

Supplement: Supplementary Figure 1 — Effect of HC-067047 on GSK1016790A-induced modulation on MMP-9 and TJ. TRPV4 antagonist HC-067047 (10 μ M/mouse) was icv. injected 30 min before GSK1016790A injection, and then HC-067047 was injected once daily for the 3 days. GSK1016790A-induced the increase of MMP-9 protein level and activity (A), and the decrease of ZO-1 and occludin protein levels (B) were blocked completely by HC-067047. **P < 0.01 vs. control and ##P < 0.01 vs. GSK1016790A-injected mice. [file Image1.TIF]
